# Bone marrow-derived cells in ocular neovascularization: contribution and mechanisms

**DOI:** 10.1007/s10456-016-9497-6

**Published:** 2016-02-15

**Authors:** Fan Gao, Huiyuan Hou, Hongliang Liang, Robert N. Weinreb, Haiyan Wang, Yusheng Wang

**Affiliations:** Department of Ophthalmology, Eye Institute of Chinese PLA, Xijing Hospital, Fourth Military Medical University, No. 127, Changle West Road, Xi’an, China; Department of Cardiovascular Surgery, Institute of Cardiovascular Disease of Chinese PLA, Xijing Hospital, Fourth Military Medical University, Xi’an, 710032 Shaanxi Province People’s Republic of China; Department of Ophthalmology, University of California, San Diego, La Jolla, CA USA

**Keywords:** Bone marrow-derived cells, Stem cells, Ocular neovascularization, Mechanism

## Abstract

Ocular neovascularization often leads to severe vision loss. The role of bone marrow-derived cells (BMCs) in the development of ocular neovascularization, and its significance, is increasingly being recognized. In this review, we discuss their contribution and the potential mechanisms that mediate the effect of BMCs on the progression of ocular neovascularization. The sequence of events by which BMCs participate in ocular neovascularization can be roughly divided into four phases, i.e., mobilization, migration, adhesion and differentiation. This process is delicately regulated and liable to be affected by multiple factors. Cytokines such as vascular endothelial growth factor, granulocyte colony-stimulating factor and erythropoietin are involved in the mobilization of BMCs. Studies have also demonstrated a key role of cytokines such as stromal cell-derived factor-1, tumor necrosis factor-α, as well as vascular endothelial growth factor, in regulating the migration of BMCs. The adhesion of BMCs is mainly regulated by vascular cell adhesion molecule-1, intercellular adhesion molecule-1 and vascular endothelial cadherin. However, the mechanisms regulating the differentiation of BMCs are largely unknown at present. In addition, BMCs secrete cytokines that interact with the microenvironment of ocular neovascularization; their contribution to ocular neovascularization, especially choroidal neovascularization, can be aggravated by several risk factors. An extensive regulatory network is thought to modulate the role of BMCs in the development of ocular neovascularization. A comprehensive understanding of the involved mechanisms will help in the development of novel therapeutic strategies related to BMCs. In this review, we have limited the discussion to the recent progress in this field, especially the research conducted at our laboratory.

## Introduction

Ocular neovascularization (NV) can occur in multiple ocular compartments, including in cornea, iris, retina, optical disc and choroid. It often leads to intractable and severe vision loss [1–3]. These new vessels have abnormal cellular components, are structurally fragile and prone to hemorrhage. The resultant hemorrhage often causes accumulation of blood in the anterior chamber and vitreous which reduces visual acuity. Moreover, the resultant edema, exudates and accompanying fibrosis may impair vision by several mechanisms including reduced corneal transparency and increased intraocular pressure that may result in structural and functional impairment of retinal neurons [3].

For years, it had been demonstrated that in addition to cells in situ, bone marrow-derived cells (BMCs) contribute to the formation of ocular neovascularization [4]. Hence, a better understanding of BMCs’ contribution to ocular neovascularization is important to the development of new therapeutic targets and strategies. The steps and mechanisms involved in BMCs’ contribution to ocular neovascularization are complex and involve multiple regulating factors, cells and pathways. We review the main mechanisms that are known to modulate the role of BMCs in ocular neovascularization, especially in corneal neovascularization (CV), retinal neovascularization (RNV) and choroidal neovascularization (CNV), and discuss the risk factors affecting the contribution of BMCs.

## Bone marrow-derived cells and ocular neovascularization

Neovascularization involves two main modalities. During angiogenesis, endothelial cells (ECs) sprout from adjacent blood vessels. With vasculogenesis, cells from circulation form blood vessels [5]. Ocular neovascularization involves both modalities, and the available evidence implicates a variety of BMCs in the pathogenesis of ocular neovascularization [6, 7].

The participation and differentiation of BMCs in neovascularization in cornea, retina and choroid has been widely studied. BMCs consist of various types of stem cells as well as circulating mononuclear macrophages [7, 8]. Recent studies suggest an important role for endothelial progenitor cells (EPCs), hematopoietic stem cells (HSCs) and mesenchymal stem cells (MSCs) in ocular neovascularization [4]. These stem cells have been shown to differentiate into different several cell types, including vascular components (vascular ECs, vascular smooth muscle cells (VSMCs), and pericytes) and extravascular cells (myofibroblasts, inflammatory cells, retinal pigment epithelial (RPE) cells and glial cells) [1]. In addition to stem cells, bone marrow (BM)-derived macrophages are key modulators of the severity of ocular neovascularization. As an example, after entering the area of neovascularization, macrophages secrete angiogenic factors and stimulate expression of stromal-derived factor-1 (SDF-1) in RPE cells, which is associated with migration and adhesion of BMCs into ischemic or injured tissue [8].

### Bone marrow-derived cells and corneal neovascularization

Several inflammatory disease states including infectious diseases and trauma are known to be associated with CV [9]. CV can be artificially induced in animal models by different approaches as an aid to investigate mechanisms of not only corneal pathology but also of general angiogenesis [10]. In a rat model of suture-induced inflammatory CV, the CD11b-positive myeloid cells appeared to pre-pattern the extracellular matrix (ECM), direct the growth of sprouts, and incorporate into the sprout tip endothelium, indicating BMCs’ participation in inflammatory CV [11]. Moreover, implantation of a Hydron pellet containing basic fibroblast cell growth factor (bFGF), in the absence of substantial inflammation, was shown to induce CV. Pericytes involved in this model of CV had a dual source, i.e., BM and preexisting limbus capillaries, whereas ECs only originated from preexisting capillaries. At sites of CV, >90 % of BM-derived pericytes were shown to express CD45 and CD11b, respectively, which indicates their hematopoietic origin from the BM, and their contribution in causing CV [6]. This finding was further supported by other studies that employed various factors for inducing CV via mobilization, migration and incorporation of BM-derived EPCs such as VEGF [12], granulocyte macrophage colony-stimulating factor (GM-CSF) [13], secretoneurin [14] and peroxisome proliferator-activated receptor-δ [15].

### Bone marrow-derived cells and retinal neovascularization

Retinal neovascularization is the main complication of proliferative vascular diseases, such as diabetic retinopathy (DR) and retinopathy of prematurity. Some common themes have been identified in different disease states. Retinal ischemia/insult is a key feature of RNV, as is the involvement of BMCs [16]. A study by Grant et al. [17] indicated that BM-derived HSCs can differentiate into all hematopoietic cell lineages involved in the pathogenesis of RNV. They also found the recruitment of EPCs at the sites of ischemic lesion [17]. Another study confirmed the involvement of BMCs, which had the characteristic elongated appearance of EC, incorporated into forming RNV by selectively targeting retinal astrocytes [18]. This finding is supported by the results of several other studies that showed the contribution of BMCs in laser injury-associated model and oxygen-induced retinopathy (OIR) model [19–25].

The involvement of BMCs has also been confirmed clinically. In surgically excised epiretinal membranes from patients with proliferative DR, the presence of BM-derived CD133^+^ EPCs and CD14^+^ monocytes has been documented, which further implicates BMCs are involved in retinal angiogenic diseases [26].

### Bone marrow-derived cells and choroidal neovascularization

Choroidal neovascularization represents the final pathway leading to severe visual loss in approximately 40 ocular diseases, of which the most common is age-related macular degeneration (AMD) [16]. Mounting evidence indicates the involvement of BMCs in CNV, both in clinical specimens and in animal models. In patients with AMD, AC133 positive cells, a putative marker of EPCs and HSCs, were detected in surgically excised CNV specimens. Immunoreactivity showed that the AC133-positive cells in the avascular fibrous stromal core were RPE cells and macrophages, and those in the highly vascular peripheral were ECs [27]. In addition, a series of clinical studies conducted in Japan indicated a correlation of circulating HSCs with neovascular AMD, idiopathic CNV and pathologic myopia [28–30]. The involvement of BMCs has also been confirmed in animal models of CNV. Espinosa-Heidmann et al. [31] were the first to report the role of BMCs in CNV in the year 2003. This was further supported by several others that showed that the recruitment of BMCs was associated with the severity of CNV and that targeting homing and adhesion of BMCs using antibody treatment may prevent CNV [32–34]. In a laser-induced mice model of CNV, we observed differentiation of BMCs into parts of vascular structure and surrounding sprouts, thus further implicating BMCs in the pathogenesis of CNV [7].

### Proportions and time frame of bone marrow-derived cells’ contribution to ocular neovascularization

In different kinds of ocular neovascularization, proportions of BMCs in the cellular vasculature are diverse. Only 17.7 % of vascular ECs and 53 % of pericytes were shown to originate from BM in CV, whereas in CNV, the proportion of BM-derived vascular ECs was approximately 50–60 % [6, 35]. In mice treated with both transplantation of green fluorescent protein-labeled BM and laser-induced CNV, the percentage of BMCs in CNV may be even greater and more stable if the documented repopulation with transplanted stem cells is >90 % because of the link between the percentage of BMCs in CNV and the repopulation in chimeric mice [31].

The time frame of BMCs’ contribution to ocular neovascularization remains understudied. It is believed that BMCs arrive at the site of ocular neovascularization at the initiation of neovascularization. In inflammatory CV, BM-derived myeloid cells were shown to form endothelium-free tunnel in response to inflammatory stimuli in the initial hours after induction; their peak levels were observed at day 1 [11]. In a mice model of CNV, the presence of BMCs was demonstrated in the lesions as early as day 3 after laser treatment. Further, using bioluminescence imaging the dynamic conduct of BMCs in CNV has been observed. The number of BMCs appeared to peak at day 7 after treatment with the numbers showing a decline from the day 14 onwards and remained stable from day 28 [7]. In addition, the continuous presence of macrophages was shown in lesions of CNV and other areas of the eye from day 7 after laser treatment [36]. However, the time frame of BMCs in RNV remains unclear and requires further investigation.

## Molecular mechanisms of bone marrow-derived cells’ contribution to ocular neovascularization

**Fig. 1:**
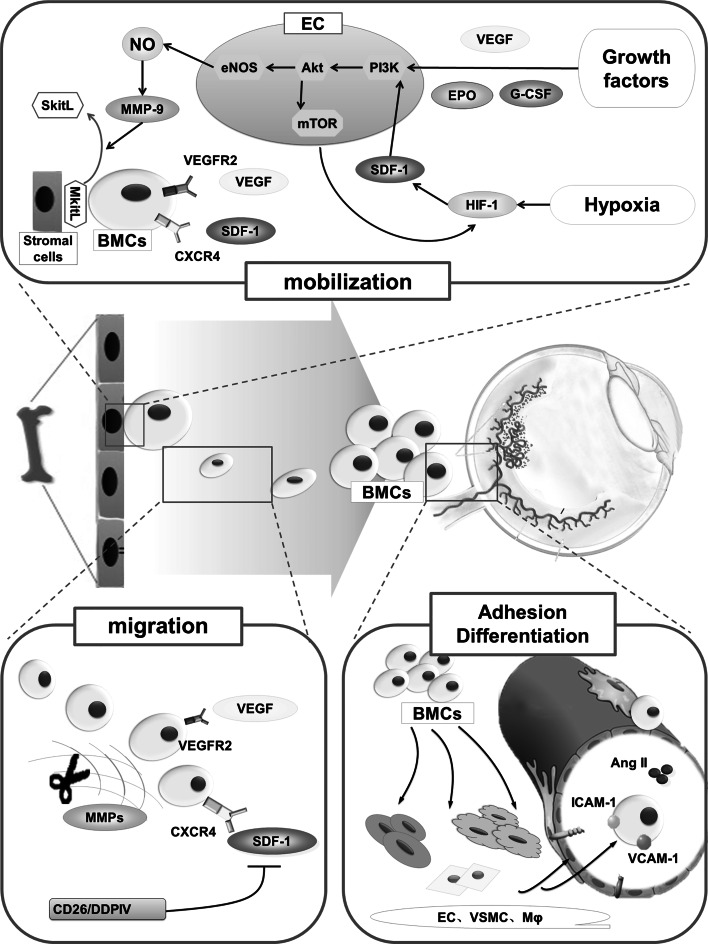
Pathways and mechanisms underlying the contribution of BMCs in the pathogenesis of ocular neovascularization. BMCs contribute to the development of ocular neovascularization in four phases (mobilization, migration, adhesion and differentiation), and this process is finely regulated by complex signaling pathways. Cytokines, such as VEGF and SDF-1, are involved in mobilization and migration; the adhesion of BMCs is mainly regulated by adhesion molecules including VCAM-1 and ICAM-1. However, the mechanisms regulating the differentiation of BMCs are largely unknown at present. *BMCs* bone marrow-derived cells, *VEGF* vascular endothelial growth factor, *VEGFR* VEGF receptor, *G-CSF* granulocyte colony-stimulating factor, *EPO* erythropoietin, *HIF-1* hypoxia-inducible factor-1, *SDF-1* stromal cell-derived factor-1, *CXCR4* CXC receptor-4, *eNOS* endothelial nitric oxide synthase, *MMPs* matrix metalloproteinases, *MkitL* membrane-bound kit ligand, *SkitL* soluble kit ligand, *EC* endothelial cells, *VSMCs* vascular smooth muscle cells, *Mφ* macrophages, *Ang II* angiotensin II, *ICAM-1* intercellular adhesion molecule-1, *VCAM-1* vascular cell adhesion molecule-1

Although BMCs in ocular neovascularization mentioned above are different in several respects (such as their proportions, time frame of BMCs’ contribution), they also share some similarities. For example, their participation in the development of ocular neovascularization includes four steps, i.e., mobilization, migration, adhesion and differentiation, and are regulated and affected by multiple factors (Fig. 1).

### Mobilization of bone marrow-derived cells from bone marrow to circulation

On stimulation by injury or drugs, BMCs are mobilized from BM to circulation to perform several physiological and pathological roles. Both preclinical studies and clinical studies have documented an increasing expression of various cytokines (such as VEGF) occurring in response to local tissue injury, both in circulation and in clinical specimens of ocular neovascularization tissues [37, 38]. As a result, the BM microenvironment transitions from a quiescent to an activated state, and the mobilization of BMCs occurs secondary to these increased levels, including those of VEGF, granulocyte colony-stimulating factor (G-CSF) and erythropoietin (EPO) levels [4].

VEGF-A, a member of VEGF family, is known to play a crucial role in the development of ocular neovascularization. In CV, the interaction of VEGF-A and VEGF receptor 1 has an immune amplification effect in facilitating neovascularization via recruitment of mononuclear macrophages [39]. Unbalanced expressions of VEGF and pigment epithelium-derived factor (PEDF) are thought to be the primary cause of ocular neovascularization. In addition to its localized angiogenic effects in the neovascular lesions, the synergistic effect of both increased VEGF-A and decreased PEDF may also have a systemic effect that blocks the mobilization of EPCs from BM to attenuate RNV [25]. VEGF, which is secreted from both ocular tissues and BMCs, can of itself contribute to the mobilization of BMCs [12, 40].

The endothelial nitric oxide synthase (eNOS) pathway plays a pivotal role in that process with both matrix metalloproteinase-9(MMP-9) and eNOS appearing to be of particular importance. In earlier studies, deletion of these genes was shown to block the mobilization of BMCs from the BM. Further, binding of VEGF to VEGF receptor-2, and that of SDF-1 to CXCR4, appeared to enhance mobilization of BMCs, resulting in the release of MMP-9 through activation of the eNOS pathway, which includes phosphorylation of Akt [12, 41]. Due to the increased MMP-9, the membrane-bound kit ligand is converted into soluble kit ligand, which weakens the interaction of stromal cell and BMCs, thereby facilitating the mobilization of BMCs from BM to peripheral blood [42].

Cytokines such as G-CSF and GM-CSF are also known to promote mobilization of the BMCs to periphery. More recently, it was shown to play a role both within the cells themselves and in the BM microenvironment, instead of the G-CSF receptors on BMCs. A recent study demonstrated the effect of G-CSF on the expression and function of HSCs surface adhesion molecules in inhibiting apoptosis of HSCs [43]. In addition, G-CSF has also been reported to decrease the expression of vascular cell adhesion molecule-1 (VCAM-1) in BM, which in turn stimulates release of MMPs and ECM degeneration. In the context of ocular neovascularization, mobilization of EPCs from BM into the circulation was shown to have occurred in response to GM-CSF in enhanced CV [13].

Erythropoietin is a hormone known to stimulate BM erythrocyte production. Increased EPO levels in post-MI heart failure were shown to increase the number of EPCs in the peripheral blood [44]. Further, EPO is also known to facilitate mobilization of BMCs into the circulation to promote ocular neovascularization. Earlier studies conducted on OIR mouse model revealed a suppression of EPO levels in the vessel loss phase. Early use of EPO could protect against hypoxia-induced retinal neuron apoptosis. Conversely, late use of EPO was shown to aggravate pathological neovascularization instead of protecting the retina, which suggests that EPO likely contributes to the mobilization and recruitment of BMCs into retinal neovascular lesions at a time when the angiogenic response has already started [45].

### Migration of bone marrow-derived cells from circulation to ocular lesions

Owing to the chemotactic effect of related cytokines, BMCs can migrate from circulation into the ocular neovascularization lesions. This process is regulated by a complex interplay between multiple pathways, the mechanism of which is not well understood.

In several studies, BMCs have shown significant chemotactic response to several factors in ocular neovascularization. A typical example is MSCs which was shown to readily migrate to neovascular area as against the other normal organs in a study of CNV [46]. In another study of RNV, the targeted migration of BMCs was shown to be related with R-cadherin. The mechanism was similar to that in the migration of endogenous retinal vascular ECs along an astrocytic template using R-cadherin-mediated guidance, during formation of retinal vascular layer and plexuses [47]. Generally, the chemokines and proinflammatory cytokines such as tumor necrosis factor-α (TNF-α) are the most critical factors in signals of migration.

Chemokines and their receptors play an important role in the selective migration of BMCs. These include SDF-1/CXCR4 and fractalkine/CX3CR1. Chemokines with chemoattractant properties belong to four families: CXC, CC, CX3C and C. SDF-1 is a member of CXC chemokine family and induces migration of BMCs to the target tissues via binding to CXCR4 expressed on various cells like vascular ECs and BMCs [48]. Further, SDF-1/CXCR4 have been implicated in causing the influx of EPCs, HSCs and MSCs in RNV and CNV models, and CXCR4 antagonists have been shown to reduce the influx of these BMCs [41]. SDF-1 upregulation can be facilitated by the hypoxic-inducible factor-1α in the injured or hypoxic RPE layer, resulting in migration of BMCs to the ocular region [48]. In addition, amyloid β in drusen was shown to enhance EPCs migration via upregulation of CX3CR1, a receptor of fractalkine, which is also known to facilitate the development of CNV [49].

Tumor necrosis factor-α was earlier believed to enhance tissue inflammation; however, in a study on CV, absence of TNF-α was shown to augment pathological neovascularization and post-inflammatory scarring instead of ameliorating the same [50]. Another study demonstrated that TNF-α receptor 1b, which is selectively expressed on BM-derived inflammatory cells, induced signals to upregulate inflammatory cell invasion and promote angiogenic response in CNV [51].

In addition to the cytokines mentioned above, ocular tissues stimulated by different factors were also shown to produce enzymes such as MMPs that degenerate ECM, promote sprouting of new blood vessels, and serve to enhance the contribution of BMCs to ocular neovascularization [52].

### Adhesion of bone marrow-derived cells in ocular neovascularization

When BMCs target sites of ocular neovascularization, many cell adhesion molecules, including VCAM-1, intercellular adhesion molecule-1 (ICAM-1) and vascular endothelial cadherin (VE-cadherin), facilitate the process of adhesion. *In vivo* studies examining the adhesion of BMCs have shown an increased expression of VE-cadherin after EPCs mobilization. Further, with the influx of BMCs in the neovascular area, the VCAM-1 and ICAM-1 expressions on adjacent retinal vessels were also upregulated [21, 23]. These findings are consistent with those from in vitro studies that have demonstrated enhanced adhesion of BMCs to vascular ECs such as VCAM-1/VLA-4 and ICAM-1/β-2 integrin interactions after binding of these adhesion molecules to their ligands.

Other in vitro studies have shown the effect of P-selectin and angiotensin II (Ang II) on the adhesive property of BMCs. MSCs bind to ECs in a P-selectin-dependent manner in vitro. However, P-selectin glycoprotein ligand 1 is not expressed by MSCs, and the main selectin ligands on MSCs are yet to be determined [53]. Ang II, an octapeptide hormone, was shown to enhance the BM-derived EPCs-matrix adhesion, which is mediated by NO through upregulation of integrin [54].

### Differentiation of bone marrow-derived cells into neovascular cells

Most of BMCs differentiate into EC, VSMCs and macrophages after arriving at the sites of ocular neovascularization. Some of them have completed differentiation process before permeating through Bruch’s membrane, even while still in the choroidal blood vessels. Interestingly, some BMCs were found in cornea, optic disc and iris in the absence of neovascularization; some of them represent arborization shape and have been shown to be F4/80-positive, which suggests that there may be other functions for BMCs in the eye besides its contribution to ocular neovascularization [7].

Different microenvironments induce differentiation of BMCs in several directions, and the mechanism of interaction of BMCs and ocular neovascularization microenvironment needs to be more clearly defined. In the microenvironment of newborn mice retinal neurons, there are cytokines which can induce BMCs differentiation and activate specific signaling pathways. Moreover, in RNV and retinal degenerative disease, BMCs implanted in retinal inferior vena have been shown to differentiate into vascular ECs and photoreceptor cells, and to exert profound vasculotrophic and neurotrophic effects [17, 18, 22, 55]. The differentiation of BMCs in CNV has also been investigated. In vivo studies have shown a concentration of BMCs, most of which are of EPCs phenotype, at the RPE layer with prominent expression of SDF-1 [48]. Moreover, in vitro experiments have shown that higher levels of SDF-1 could induce differentiation of BMCs into ECs (unpublished). Indeed, the SDF-1/CXCR4 signaling pathway may not only contribute to the chemotactic effect, but also may induce BMCs differentiation into EC, which is necessary for the development of ocular neovascularization.

As the ocular neovascularization is developing, the ECs differentiate into tip and stalk cells, a process that is regulated by Notch signaling [56]. Recent studies found that notch signal could not only suppress or stimulate the tip cell formation, but also affect EPCs mobilization, proliferation and differentiation. The role of Notch signaling is complex and varies in different kinds of neovascularization. In some kinds, it stimulates neovascularization, while in some others it suppresses it or causes vascular regression partly via regulating the differentiation and functionality of EPCs [57–59]. In ischemic neovascularization, Jagged-1, one of the Notch ligands, was found to mediate notch signals for stimulating differentiation of EPCs toward the endothelial lineage, which appeared to enhance neovascularization [58]. Another study on a mice model of CNV showed that deficiency of recombination signal-binding protein Jκ (RBP-J), the transcription factor downstream of notch receptors, induces a more intensive angiogenic response to injury and may modulate differentiation of EPCs into circulating ECs. Further, RBP-J deficiency was also shown to induce angiogenesis in retina and cornea, which suggests that the RBP-J-mediated notch signaling may be involved in the maintenance of the vascular homeostasis in eye, partly by affecting EPCs differentiation [59].

## Cytokines from bone marrow-derived cells

BMCs not only take apart in the formation of vascular structure, but are also known to secret multiple kinds of cytokines that effect vascular cells and the surrounding tissues in addition to BMCs themselves, thus regulating the development of ocular neovascularization. VEGF, bFGF, SDF-1, cathepsin L, MMP-13 and plasminogen activator inhibitor (PAI-1) are all known to be produced by BMCs [4, 40]. It is also known that BM-derived MSCs secrete most of the pro-angiogenic factors necessary for ocular neovascularization (such as, VEGF). Under hypoxic conditions, MSCs were shown to migrate into ocular lesions and produce VEGF through paracrine mechanism to stimulate vascular ECs proliferation and further influx of BMCs [46, 60]. Others have also reported that BMCs in CNV lesions are a rich source of VEGF and bFGF [7].

Besides sufficient pro-angiogenic factors, the degradation of basement membranes and extracellular matrix is a precondition for sprouting of vascular cells and the neovascularization. Proteolytic enzymes such as cathepsin cysteine proteases and MMPs are known to induce ocular matrix degradation leading to ocular neovascularization. In both OIR and CNV models, for the most part, cathepsin L in lesions was from EPCs, which are VE-cadherin-positive and have been shown to contribute to ocular neovascularization. Both in wild-type mice with cathepsin L inhibitors and in cathepsin L-deficient mice, a similar decrease in ocular neovascularization has been documented. CNV-related in vitro and in vivo studies have demonstrated that the pro-angiogenic effect of MMP-13 is mainly mediated through BM-derived MSCs. In the event of excessive secretion of MMP-13 by MSCs, the state of hemostasis between stimulatory and inhibitory angiogenic factors may be disrupted, thereby stimulating CNV [52].

Recent studies have revealed that the same cytokines may contribute to neovascularization by different mechanisms, depending upon their origin. For example, PAI-1 derived from BMCs, as against that derived from host ocular cells, plays a crucial role in the development of CNV. Interestingly, a contrasting phenomenon is demonstrable in tumor-related neovascularization in which PAI-1 produced by host cells was shown to play a key role [61].

## MicroRNAs in bone marrow-derived cells’ contribution to ocular neovascularization

MicroRNAs (miRNA) are endogenous small (19–22 nucleotides), non-coding RNAs generated by the sequential processing of primary miRNA and Dicer1 in the cytoplasm. Mature miRNAs associate with the 3′ untranslated regions of the specific target mRNAs to downregulate gene expression by targeting mRNAs for translational suppression or mRNA degradation [62].

Furthermore, recent studies also indicated that miRNAs were shown to regulate the development of RNV and CNV, which BMCs are involved in [63]. MicroRNA-126 is one of the most extensively studied microRNAs known to be specifically expressed in ECs and HSCs [64, 65]. A downregulation of miR-126 has been observed in the retina from OIR mice as well as in hypoxic cultures of chorioretinal vessel ECs [66, 67]. Moreover, an overexpression of miR-126 was shown to reduce RNV by targeting VEGF, hypoxia-inducible factor-1α and insulin-like growth factor [66].

VEGF, an important stimulator of neovascularization in several tissue including ocular neovascularization, is not only a target protein of several miRNAs such as miR-126 and miR-200b, but was also shown to induce expression of miRNAs like miR-132 in ECs [68–71]. Other miRNAs, including miRNA-31, -150 and -184, which are decreased in RNV and CNV, were also shown to be involved in mediating neovascularization via their regulatory effect on different signaling proteins [72] (Table 1).

**Table 1:** Non-exhaustive list of microRNAs implicated in ocular neovascularization

MicroRNA	Target genes	Type of ocular neovascularization	References
MiR-126	VEGF, HIF-1a, IGF	RNV, CNV	[65–68]
MiR-200b	VEGF, OXR1	RNV	[69, 70]
MiR-31	PDGF-B, HIF-1α	RNV, CNV	[72]
MiR-150	VEGF, PDGF-B	RNV, CNV	[72]
MiR-184	Fzd4	RNV, CNV	[72]
MiR-132	Ras-GAP	CV	[71]

*MiR* microRNA, *HIF-1α* hypoxia-inducible factor-1α, *IGF* insulin-like growth factor, *OXR1* oxidation resistance 1, *PDGF-B* platelet-derived growth factor-B, *Fzd4* Frizzled4, *Ras-GAP* Ras-glyceraldehyde-3-phosphate, *RNV* retinal neovascularization, *CNV* choroidal neovascularization, *CV* corneal neovascularization

Despite more and more miRNAs that are now known to be expressed in vascular cells of ocular neovascularization, and the delineation of the roles of some of these miRNAs in BMCs, the specific expression and functionality of the majority of miRNAs in BMCs that contribute to ocular neovascularization have not been elucidated [16]. MiRNAs and their target genes involved in this event are yet to be discovered, and a growing body of evidence supporting the effect of miRNAs presents a potential strategy.

**Fig. 2:**
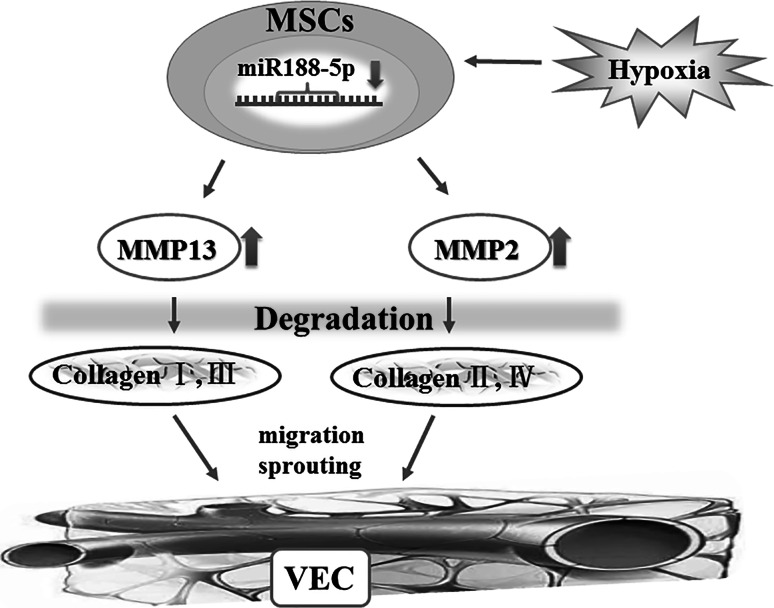
Role of miR-188-5p in regulating the involvement of BMCs in the pathogenesis of choroidal neovascularization. We observed the relationship between miR-188-5p and MMP-2/13 in MSCs and demonstrated that overexpression of miR-188-5p attenuated the development of CNV as well as the effect of BMCs (unpublished data). These findings are consistent with the hypothesis that miR-188-5p may affect the proliferation, migration and apoptosis of vascular cells and fibrosis of CNV via regulating the MMP-2 and -13 expressions in BMCs, especially MSCs. *BMCs* bone marrow-derived cells, *MSCs* mesenchymal stem cells, *MMP2/13* matrix metalloproteinase-2/13, *VEC* vascular endothelial cells

Our group has found previously that hypoxia significantly downregulated the expression of miR-188-5p and upregulated expressions of MMP-2 and -13 in BM-derived MSCs of mice (unpublished). To investigate the role of miR-188-5p, and its possible target genes that are related to BMCs in CNV, we propose that miR-188-5p may regulate degradation of ECM and PEDF in CNV, which in turn affects the proliferation, migration and apoptosis of vascular cells and fibrosis of CNV via regulation MMP-2 and -13 expressions. Further, the relation between miRNA and BMCs in CNV has been described (Fig. 2).

## Risk factors affecting bone marrow-derived cells’ contribution to ocular neovascularization

It is noteworthy that some risk factors for neovascular diseases such as aging and smoking tend to alter the BM microenvironment leading to exhaustion of BMCs and their contribution to the development of neovascularization. The effects of risk factors on ocular neovascularization, especially CNV, have also been documented.

### Aging

BMCs are affected by several risk factors like aging and are known to regulate the severity of ocular neovascularization formation. Aging BMCs are known to have age-related dysfunction, which may be associated with a more intense response to pro-vascularization factors, an increased number of cells distributed to their corresponding positions and an altered direction of differentiation, thereby enhancing the severity of ocular neovascularization [1]. In their earlier studies of CNV, Espinosa-Heidmann et al. reported the effect of aging on neovascular remodeling [73, 74]. Recent work at our laboratory using in vivo bioluminescence imaging also suggested that aging BMCs aggravate the severity of CNV (unpublished). A study focusing on BMCs also demonstrated that the severity of CNV is more associated with the age of the cells within the BM, rather than with the age of the retina or choroid [75]. Another study demonstrated that HSCs showed more myelocytes in aged mice as compared to that in the young, which altered the balance of differentiation of HSCs toward lymphoid and myeloid lineage. The age-related function in BMCs appears to be related to the aberrant expression of some cytokines, growth factor receptors and cellular adhesion molecules [76]. Among them, aging appeared to cause Fasl gene change in eye, which induced the migration of pro-angiogenic M2 macrophages into the laser-induced lesions and increased pro-angiogenic cytokines promoting CNV [76, 77].

### Smoking

Smoking, one of the most important risk factors for AMD, can serve to promote participation of the BMCs in the development of CNV. Nicotine, the major component of the particulate phase of cigarette smoke, was shown to induce an increase in the severity of CNV in a mouse model [74]. This finding is supported by another study in which nicotine-induced VEGF release, mediated through nicotinic acetylcholine receptor in VSMCs, was the likely underlying mechanism for the contribution of VEGF toward neovascularization in cigarette smokers. This process was shown to be mediated by epidermal growth factor receptor-extracellular signal-regulated kinase pathway [78]. Consistent with earlier studies, we have earlier shown that nicotine promotes recruitment and incorporation of BMCs into CNV and affects differentiation of BMCs in CNV, which is partly due to upregulation of VEGF, bFGF and VCAM-1 [79]. The one common feature in these studies is the angiogenic stimulating factor, VEGF. This commonality suggests an indirect effect of nicotine on BMCs’ contribution to CNV, possibly mediated via other factors. However, more mechanistic studies need to be conducted in the future.

### Hyperglycemia

Hyperglycemia has also been recognized as a risk factor for ocular neovascularization and vision loss. DR is the most common cause of vision loss among 20- to 74-year-old Americans [80]. Our group focused on CNV has documented very high numbers of BMCs contributing to lesions in a mice model of CNV and demonstrated greater recruitment and incorporation of BMCs into CNV in mice with diabetes mellitus [7, 60]. We also found an increase in the ratio of BMCs expressing ECs marker or macrophage marker, besides upregulated expression of VEGF and SDF-1 in CNV in vivo. In addition, human MSCs migration and expression of VEGF and SDF-1 in RPE cells appeared to increase on (in vitro) culture in high concentration of glucose [60]. This implies that hyperglycemia enhanced the expression of VEGF and SDF-1 in RPE cells, promoted recruitment and incorporation of BMCs, and affected differentiation of BMCs in CNV which appeared to increase the severity of CNV in diabetic mice. Hyperglycemia-induced oxidative stress is an upstream factor that promotes STAT3 activity, leading to the activation of VEGF transcription, which may indirectly accelerate the participation of BMCs and eventually exacerbate CNV [81].

## Research needs and future research priorities

As discussed in this review, BMCs appear to play key roles in the development of ocular neovascularization with implications for the present anti-VEGF therapy as well as for the future antiangiogenic therapies. However, the regression of ocular neovascularization, such as RNV and CNV, is rarely permanent and the regrowth of new vessels is observed in a few months without multiple applications of anti-VEGF agents [82]. To date, few studies have investigated the function and mechanisms underlying the role of BMCs in the recurrence of ocular neovascularization. The answers to this and other questions may help in formulating a combination of targeting BMCs therapy and current therapies to help optimize future antiangiogenic treatment.

In addition, the diagnosis of ocular neovascularization is often established only after formation of new vessels, or sometimes even with accompanying exudates and hemorrhage. It is now evident that BMCs arrive at the lesion prior to the formation of new vessels after their mobilization from BM and migration to the peripheral circulation. This makes it feasible to detect the activity of BMCs in the patients’ blood before the solid mass formation. Sasahara et al. provided convincing evidence of the abnormal number and function of circulating BMCs in patients of CNV, as being one of the systemic factors [28–30]. These studies led us to hypothesize whether BMCs and related cell factors could serve as potential markers to help in early detection of ocular neovascularization or recurrence of ocular neovascularization. Further prospective and longitudinal studies are still required.

Overall, it is still a largely unexplored field which can potentially help improve our understanding of the molecular mechanisms underlying the effect of BMCs in ocular neovascularization, and extend the clinical application of BMCs and their therapeutic use in the future.

## Summary

Mounting evidence indicates that BMCs play a pivotal role in the pathogenesis of ocular neovascularization, which can be roughly divided into four steps, mobilization, migration, adhesion and differentiation. Each step is regulated by multiple factors. Cytokines mediating the BMCs’ contribution, such as VEGF, G-CSF, EPO, SDF-1, TNF-α, VCAM-1, ICAM-1, VE-cadherin, have been identified and represent potential novel targets for therapeutic application of BMCs. Furthermore, the identified risk factors for participation of BMCs in ocular neovascularization offer other potential targets for neovascularization interventions. A better understanding of the mechanisms underlying the involvement of BMCs in different kinds of ocular neovascularization will help to optimize ocular antiangiogenic therapy. Besides, this is an essential prerequisite for the use of BMCs as targets or vehicles for application not only to the eye but also to other parts of the body.

## Abbreviations

AMD: Age-related macular degeneration
bFGF: Basic fibroblast cell growth factor
BM: Bone marrow
BMCs: Bone marrow-derived cells
CNV: Choroidal neovascularization
CV: Corneal neovascularization
DR: Diabetic retinopathy
ECs: Endothelial cells
ECM: Extracellular matrix
eNOS: Endothelial nitric oxide synthase
EPCs: Endothelial progenitor cells
EPO: Erythropoietin
G-CSF: Granulocyte colony-stimulating factor
GM-CSF: Granulocyte macrophage colony-stimulating factor
HSCs: Hematopoietic stem cells
ICAM-1: Intercellular adhesion molecule-1
miRNA: MicroRNAs
MMP-9: Matrix metalloproteinase-9
MSCs: Mesenchymal stem cells
OIR: Oxygen-induced retinopathy
PAI-1: Plasminogen activator inhibitor
PEDF: Pigment epithelium-derived factor
RBP-J: Recombination signal-binding protein Jκ
RNV: Retinal neovascularization
RPE: Retinal pigment epithelial
SDF-1: Stromal-derived factor-1
TNF-α: Tumor necrosis factor-α
VCAM-1: Vascular cell adhesion molecule-1
VE-cadherin: Vascular endothelial cadherin
VSMCs: Vascular smooth muscle cells
